# MATHLA: a robust framework for HLA-peptide binding prediction integrating bidirectional LSTM and multiple head attention mechanism

**DOI:** 10.1186/s12859-020-03946-z

**Published:** 2021-01-06

**Authors:** Yilin Ye, Jian Wang, Yunwan Xu, Yi Wang, Youdong Pan, Qi Song, Xing Liu, Ji Wan

**Affiliations:** 1 Shenzhen Neocura Biotechnology Co. Ltd., Shenzhen, 518055 China; 2 School of Computer Science and Technology, Heilongjiang University, Harbin, 150080 China; 3 The Center for Microbes, Development and Health, Key Laboratory of Molecular Virology and Immunology, Institut Pasteur of Shanghai, Chinese Academy of Sciences, Shanghai, 200031 China

**Keywords:** Deep learning, HLA-peptide binding prediction, Cancer immunotherapy

## Abstract

**Background:**

Accurate prediction of binding between class I human leukocyte antigen (HLA) and neoepitope is critical for target identification within personalized T-cell based immunotherapy. Many recent prediction tools developed upon the deep learning algorithms and mass spectrometry data have indeed showed improvement on the average predicting power for class I HLA-peptide interaction. However, their prediction performances show great variability over individual HLA alleles and peptides with different lengths, which is particularly the case for HLA-C alleles due to the limited amount of experimental data. To meet the increasing demand for attaining the most accurate HLA-peptide binding prediction for individual patient in the real-world clinical studies, more advanced deep learning framework with higher prediction accuracy for HLA-C alleles and longer peptides is highly desirable.

**Results:**

We present a pan-allele HLA-peptide binding prediction framework—MATHLA which integrates bi-directional long short-term memory network and multiple head attention mechanism. This model achieves better prediction accuracy in both fivefold cross-validation test and independent test dataset. In addition, this model is superior over existing tools regarding to the prediction accuracy for longer ligand ranging from 11 to 15 amino acids. Moreover, our model also shows a significant improvement for HLA-C-peptide-binding prediction. By investigating multiple-head attention weight scores, we depicted possible interaction patterns between three HLA I supergroups and their cognate peptides.

**Conclusion:**

Our method demonstrates the necessity of further development of deep learning algorithm in improving and interpreting HLA-peptide binding prediction in parallel to increasing the amount of high-quality HLA ligandome data.

## Background

HLA-peptide binding is crucial for epitope presentation on the human cell surface and the elicitation of subsequent T cell immune response. In silico prediction of binary HLA-epitope binding or HLA-epitope binding affinity score has become one of the most essential criteria in target identification for a variety of applications in the immunotherapy [1, 2]. In general, the HLA-epitope prediction is largely dependent on state-of-the-art machine learning algorithms and substantial amount of data quantitating in vitro or in vivo HLA-epitope binding. In the past several years, a variety of deep learning models such as deep neural network [3], convolutional neural network [4] and recurrent neural network [5] have been developed for advancing HLA-peptide prediction over traditional machine learning algorithms [6]. Meanwhile, some studies weighted greatly on the data quality for improving prediction accuracy. For example, MHCflurry [3] constructed multiple models using the same architecture and discovered that the model derived from large-scale mass spectrometry data was able to outperform competing models. Recently, larger mass spectrometry data for 95 HLA class I alleles was generated, which further illustrated improving prediction accuracy by incorporating more data into the model [7]. However, data-dependent methods are confined by the limited number of alleles current technology can process and imbalance of data entries between three HLA class I supergroups (A, B, and C). As a result, data-dependent methods tend to result in lower prediction accuracy for HLA-C alleles due to smaller number of identified HLA-C ligands. Therefore, further refinement in deep learning architecture is required, which is especially the case for enhanced accuracy of pan-allele prediction tools. In contrast to the specialized model built by significant amount of training data of a fixed allele, pan-allele model is universal for predicting interaction between peptides and any HLA alleles. The principle of pan-allele model underlies that core sequences of HLA alleles can be explicitly outlined [8]. Thus, as HLA ligand sequence, the core HLA sequences can be encoded and fed into the learning algorithm for modeling HLA-peptide interaction. Compared to allele-specific methods, the pan-allele methods such as netMHCpan 4.0 [9] are more compelling to general users through higher compliance while retaining comparable performance [6]. However, prediction accuracy for a portion of HLA alleles, especially HLA-C alleles, are consistently far below average performance [6, 10]. In addition, since most natural ligands of HLAs are 8–11 amino acids in length [11], the limited amount of ligands of other lengths in the training dataset will affect the accuracy for predicting HLA-bound 12mer to 15mer [12]. Therefore, it is highly desirable to establish a more robust pan-allele model that is able to predict ligands of longer lengths and of cognate peptides of HLA-C alleles with higher accuracy.

Here we propose a novel deep learning HLA-epitope binding prediction method which takes advantage of the intrinsic ability of bi-directional LSTM to extract information from longer sequence and the ability of multiple head attention mechanism to capture contextual dependence from different angles. The proposed framework is more powerful in predicting the binding between HLA-C alleles and peptides. In addition, this framework is more robust than existing tools in predicting ligands ranging from 12 to 15 amino acids in length. Finally, this model can also help interpret the interaction between HLA alleles and peptides through multiple subspaces of interaction representation.

## Methods

### Datasets

Our training dataset is composed of data from IEDB [13], BD2013 dataset [14] and SysteMHC Atlas [15]. We only retained HLA class I ligands as well as peptides without post-translational modifications and ambiguous amino acids. In addition, ligands with relatively low confidence (prob < 0.99) in SysteMHC Atlas were excluded. All peptides in the final training dataset were between 8 and 15 amino acids in length. To balance the number between positive and negative datasets, we retrieved a companion decoy peptide from the host protein of the positive peptide from mass spectrometry data.

To convert qualitative affinity data to quantitative values, we applied a similar rule as MHCflurry [3]: positive-high, < 100 nM; positive, < 500 nM, positive-intermediate, < 1000 nM; positive-low, < 5000 nM; negative, > 5000 nM; MS-identified ligands, < 500 nM; decoys, > 5000 nM. In addition, we applied another set of rules to remove measurement redundancy for the same allele-peptide pair: keep the only data with “=” if other data were measured by inequality; if all the data are measured by “>”, the one with the greatest affinity values was retained; if all the data are measured by “<”, the one with lowest affinity value was then retained; and all the remaining data with contradictory measurements were discarded.

To facilitate model training, we normalized the original nanomolar affinity between 0 and 1.

1 $$a_{normal} = 1 - \log_{50000} \left( {a_{nM} } \right)$$

where *a*_*normal*_ is normalized affinity and *a*_*nM*_ is the original nanomolar affinity value. The final training dataset is composed of 753,961 entries for 167 HLA class I alleles (53 HLA-As, 92 HLA-Bs and 22 HLA-Cs).

The positive data of the test dataset was compiled from a recent large-scale HLA class I ligandome data covering 95 HLA alleles. Data entries from 16 HLA alleles which were previously generated by the same group [16] and were included in the training dataset were first excluded. Next, we retained HLA-displayed ligands with length of 8 to 15 amino acids and removed those with post-translational modifications. To introduce negative data into the test dataset, we randomly sampled decoy peptide sequences, which were not included in the positive datasets from the host protein-coding transcripts of the positive peptides. For each positive peptide, 100 decoy sequences were generated correspondingly. Finally, after filtering out data entries overlapping with the training dataset, there are in total 140,232 positive peptides and 13,939,114 negative decoys in the test dataset.

### Model structure

**Fig. 1:**
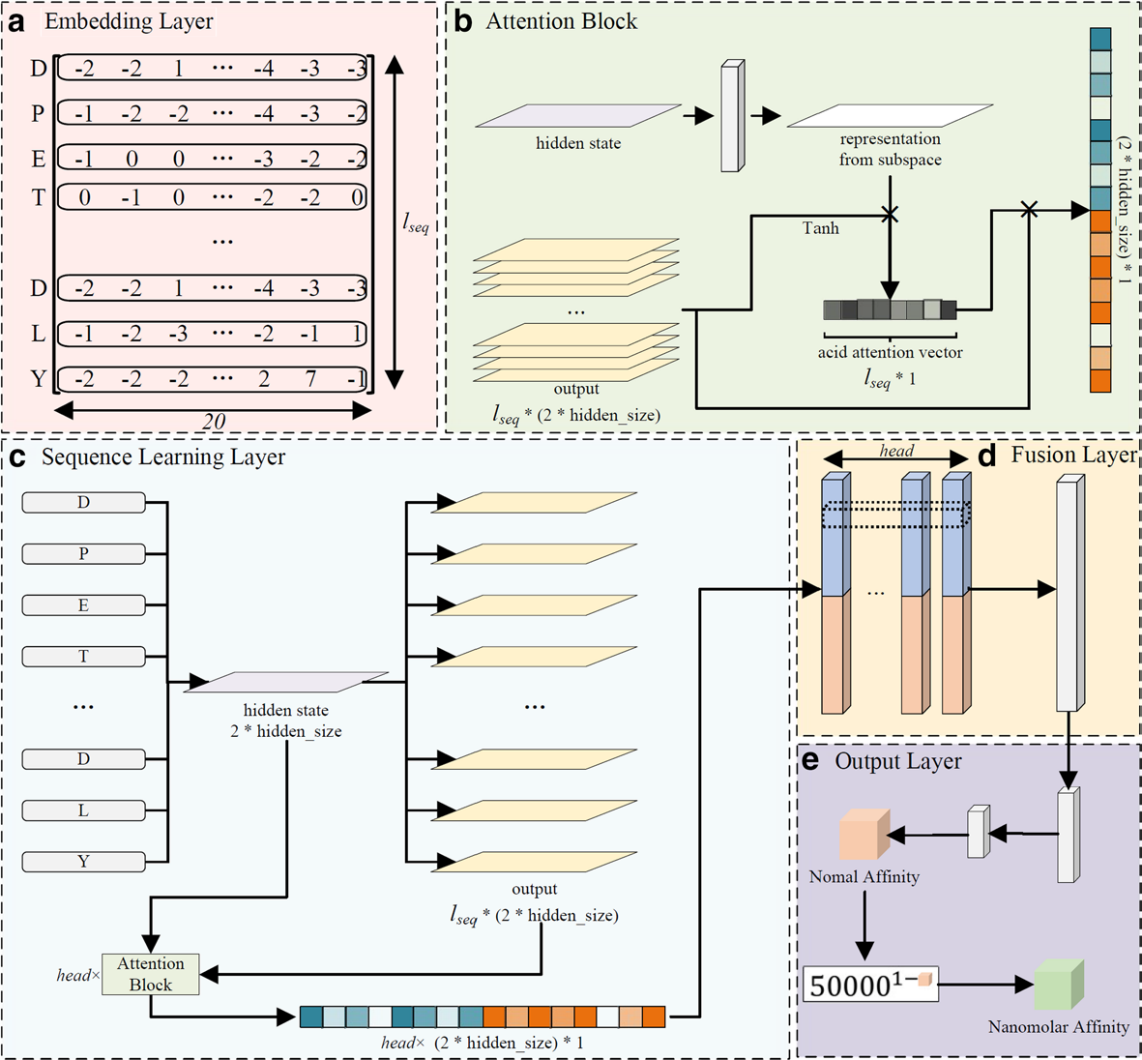
The network structure of MATHLA. **a** Embedding layer. Encode peptide and HLA pseudo-sequence through BLOSUM62 similarity matrix. **b** Sequence learning layer. Encoded information from embedding layer was input into sequence learning layer for retrieving contextual sequence features. **c** Attention block. Each head assigns weights to individual positions of the original input according to the corresponding subspace of sequence representation. **d** Fusion layer. A 2-dimension convolutional neural network with a 1*1*head filter is used to fuse vectors output from (**c**). **e** Output layer. Output normal affinity score between 0 and 1 through linear layer and sigmoid function

Each residue of peptide and HLA pseudo-sequence (retrieved from netMHCpan 4.0 [9]) is encoded to a similarity score vector according to the BLOSUM62 substitution matrix [17]. Different from many other methods with a predefined “padding” rule to ensure the equal dimension of input matrix during training, our model allows input sequences with flexible lengths. The encoded matrix with dimension *l*_seq_*20, where *l*_*seq*_ is the length of concatenated sequence of peptide and HLA pseudo-sequence, is then input into sequence learning layer (Fig. 1a).

We chose the long short-term memory network [18] to model dependence between amino acid residues of peptides with flexible lengths. Compared to conventional recurrent neural network, LSTM network bearing gate control units (input gate, forget gate, and output gate) is able to learn dependency information between distant residues within peptide sequences more effectively.

To enhance the capability of our model to learn bidirectional dependence between n-terminal and c-terminal amino acid residues, bidirectional LSTM (bi-LSTM) was used [19]. By inputting both the forward and reverse sequences to LSTM networks with the same structure respectively, the outputs of LSTM $h_{t }$ and $h_{t}^{^{\prime}}$ at time t for forward and reverse sequence are derived and the HLA-peptide sequence at position t is represented as $hidden_{t} = \left[ {h_{t} ,h_{t}^{^{\prime}} } \right]$. Finally, the output of bi-LSTM is denoted as $out^{lstm}$ (Fig. 1b).

To attend to peptide information at different positions from various subspaces of sequence representation, we applied multiple-head attention mechanism [20, 21] to the output of bidirectional LSTM.

2 $$W_{i}^{atten} = hidden^{lstm} \cdot W_{i}^{project}$$

3 $$Context_{i} = W_{i}^{atten} \cdot \left( {\tan h(out^{lstm} } \right))^{T}$$

4 $$Head_{i} = \frac{{Context_{i} }}{{\mathop \sum \nolimits_{{k{ = 0}}}^{h} Context_{k} }} \cdot out^{lstm}$$

where $hidden^{lstm} \in R^{{1 \times \left( {hidden \times 2} \right)}}$ is a hidden state of the bi-LSTM network, $W_{i}^{project} \in R^{{\left( {hidden \times 2} \right) \times \left( {hidden \times 2} \right)}}$ are weights for projecting original hidden states to different representation subspaces. $W_{j}^{atten} \in R^{{1 \times \left( {hidden \times 2} \right)}}$ are attention weights, $\left( \cdot \right)^{{\text{T}}}$ represents transpose of a matrix. $Context_{j} \in R^{{1*l_{seq} }}$ is the context vector. $out^{lstm} \in R^{{l_{seq} \times \left( {hidden \times 2} \right)}}$ is the output of LSTM network and finally $Head_{j} \in R^{{1 \times \left( {hidden \times 2} \right)}}$ is the attention vector of the original sequence under the *i*th attention mechanism (Fig. 1c).

The concatenated output vector of forward and backward attention is combined. A 2-dimension convolutional neural network (2D CNN) with a head*1*1 filter is then applied to the combined vectors for a fusion vector by learning the weight of each head of attention (Fig. 1d).

5 $$Fusion = {\text{tanh}}\left( {\left[ {Head_{1} ,Head_{2} {,} \ldots {,}Head_{h} } \right] \cdot W_{F} } \right)$$

where *h* stands for the number of head of multiple head attention module, $W_{F} \in {\text{R}}^{h \times 1 \times 1}$ is the filter of 2D CNN, and $Fusion \in R^{{1 \times \left( {hidden \times 2} \right)}}$ is the output vector after applying 2D CNN. Finally, a predicted value ranging from 0 to 1 is output by applying a linear layer with sigmoid activation function (Fig. 1e).

6 $$Output = sigmoid(Fusion \cdot W_{o} + b)$$

where $W_{o} \in R^{{\left( {hidden \times 2} \right) \times 1}}$ and *b* are the weight vector and bias for linear layer, respectively.

We randomly sampled 70% of the positive and negative data respectively from the training dataset to compile the training data. The remaining data were used as validation dataset for tuning hyperparameters.

To minimize the influence of outliers (noise) on model training, we employ an optimized Huber loss function [22] during training.

7 $$L\left( {y,\overline{y}} \right) = \left\{ {\begin{array}{*{20}l} {0.5 \times diff^{2} {,}} \hfill & {\left| {diff} \right| \le \delta } \hfill \\ {\delta \cdot \left( {\left| {diff} \right| - 0.5 \times \delta } \right){,}} \hfill & {\left| {diff} \right| > \delta } \hfill \\ \end{array} } \right.$$

8 $$diff = \left\{ {\begin{array}{*{20}l} {\min (\overline{y} - y,0),} \hfill & {if\;measurement\;is\;( < )} \hfill \\ {\max (\overline{y} - y,0),} \hfill & {if\;measurement\;is\;( > )} \hfill \\ {y - \overline{y},} \hfill & {if\;measurement\;is\;\left( = \right)} \hfill \\ \end{array} } \right.$$

where $\overline{y}$ and $y$ are values of observed and predicted binding affinity respectively. When the inequality relationship between $\overline{y}$ and $y$ is not met, their difference (*diff*) will thus affect loss. Moreover, Huber loss will degenerate to MSE loss when *diff* is less than expected value $\delta$. Otherwise Huber loss uses linear errors to evaluate training losses, which is able to minimize the impact of hard-to-learn data on the performance of model training. RAdam is used for the optimization of model parameters. Compared to traditional Adam, RAdam [23] is able to adjust the variance of adaptive learning rate so as to prevent model from converging to the local minimum. The batch size is chosen as 512 and the training stops as the loss over validation dataset shows no improvement after 5 consecutive epochs. The epoch number is set as 100. The learning rate is set as 0.001, and the dropout rate is set as 0.1.

## Results

### Model evaluation based on fivefold cross-validation

**Fig. 2:**
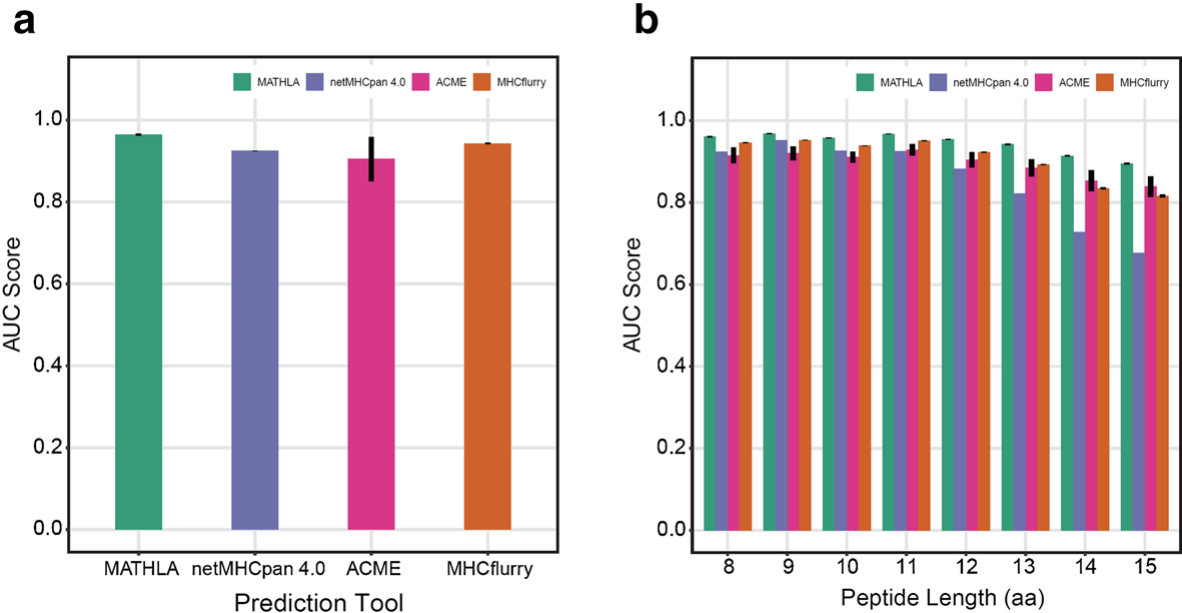
Model evaluation by the fivefold cross-validation test. **a** Fivefold cross-validation test was repeated for 10 times. The mean and standard deviation of AUC scores were shown. **b** In the fivefold cross-validation test, data in each fold were stratified by peptide length. The AUC score for each length was then calculated. The mean and standard deviation of AUC scores of 10 repeated tests were shown

To evaluate model performance and robustness, we conducted fivefold cross-validation test over training dataset. Area under the receiver operating characteristic curve (AUC) was used for model evaluation as well as model comparison against top-performed allele-specific and pan-allele models—MHCflurry, netMHCpan and ACME [24]. To ensure proper ratio of positive data to negative data in every fold of cross-validation, we separately divide positive and negative data into five individual subsets by random sampling. In each fold of cross-validation, four positive subsets and four negative subsets were pooled as training dataset whereas the remaining data were used as test dataset. The cross-validation tests were repeated for 10 times in order to calculate the mean and standard deviation. MATHLA achieves the best mean AUC score of 0.964, comparing to 0.945, 0.925 and 0.905 for netMHCpan 4.0, MHCflurry and ACME respectively (*p* values: 2.66e−16, 9.06e−12 and 4.91e−09 respectively, one-sided t-test over AUC scores of 10 cross-validation repeats) (Fig. 2a).

### The performance of MATHLA is more robust for longer HLA ligands

Most of previous methods tend to use sequence padding to handle flexible peptide lengths. Since LSTM is intrinsically designed for modelling longer sequence, we also examined the prediction performances of different tools over peptides ranging from 8 to 15 amino acids in length by fivefold cross-validation. The degrees of improvement by MATHLA over other models are positively correlated with the length of peptides (Fig. 2b). Especially for longer peptides of 12 to 15 amino acids in length, MATHLA achieves an average AUC score of 0.926 and shows 6.4%, 6.8% and 19.1% improvement of average AUC score over ACME, MHCflurry and netMHCpan 4.0. In summary, the fivefold cross-validation results demonstrate that MATHLA outperforms state-of-the-art tools in both of model performance and robustness over variable lengths of ligands.

### MATHLA outperforms existing pan-allele models on novel alleles

**Fig. 3:**
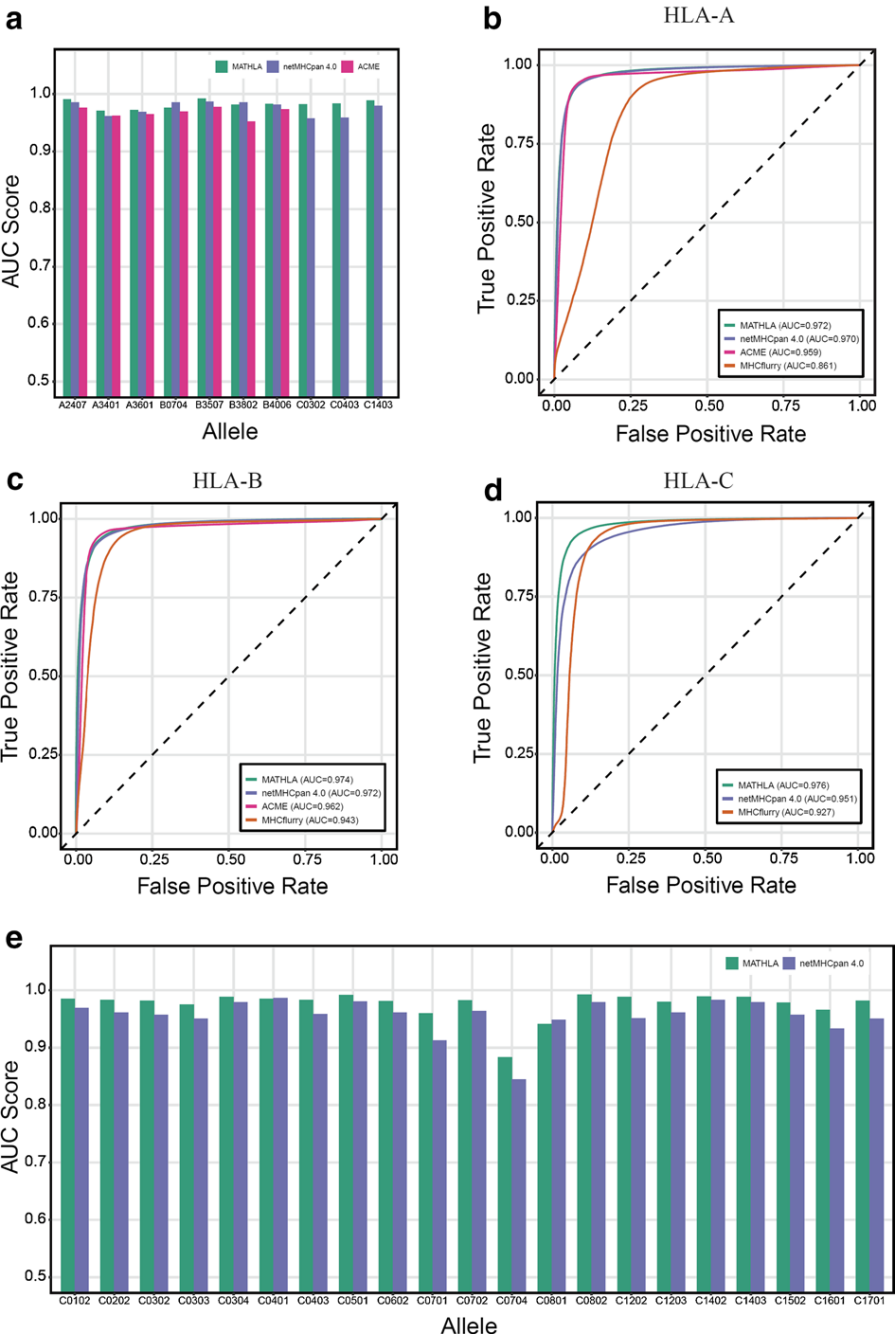
**a** AUC scores of three pan-allele models (MATHLA, netMHCpan 4.0 and ACME) over 10 non-overlapping alleles between the training and test datasets. **b–d** The receiver operating characteristic curve of MATHLA, netMHCpan 4.0 and MHCflurry on the test dataset. Plots for HLA-A, HLA-B and HLA-C alleles are generated separately. **e** The AUC scores of MATHLA and netMHCpan 4.0 over 21 HLA-C alleles in the test dataset

The foremost characteristic of a pan-allele model underlies its ability to accurately predict peptides bound to HLA alleles beyond the training dataset. To test the generalizability of MATHLA, we took the advantage of non-overlapping alleles between our training dataset and a set of mass spectrometry HLA ligandome data [7]. In total, 10 out of 95 alleles were used for assessing pan-allele model generalizability. Another two pan-allele models—netMHCpan 4.0 and ACME were used for model comparison regardless of whether these 10 alleles were included in their training datasets or not (only 7 HLA-A and HLA-B alleles are supported by ACME). In total, MATHLA outperforms netMHCpan 4.0 and ACME over 80% and 100% of non-overlapping alleles respectively. The average AUC of our model over 10 alleles reaches up to 0.982 which is higher than 0.975 of netMHCpan 4.0 (Fig. 3a). Relative to netMHCpan 4.0, it is noteworthy that performance advantage of MATHLA is more prominent for HLA-C alleles rather than HLA-A and HLA-B alleles. The average AUC score of three HLA-C alleles is 0.988 for MATHLA as compared to 0.965 for netMHCpan 4.0.

### MATHLA improves accuracy over existing models for HLA-C alleles

Inspired by the observation that MATHLA shows exceptional improvement for non-overlapping HLA-C alleles, we further compared the performance of our model over different supertypes of HLA I molecules. We separately calculated AUC scores of our model over all the HLA-A, B and C alleles in the test dataset. Pan-allele methods netMHCpan 4.0 and ACME, as well as allele-specific model MHCflurry, were used for model comparison. Although the AUC of MATHLA shows marginal enhancement over the top performed competing method netMHCpan for HLA-A and -B alleles, the AUC of MATHLA corresponding to HLA-C group is significantly improved over the competing models (0.976 for MATHLA, 0.951 for netMHCpan 4.0 and 0.927 for MHCflurry) (Fig. 3c). Moreover, we find that our model outcompetes netMHCpan 4.0 for 19 out of 21 (90.5%) individual HLA-C alleles. Since there are fewer data of HLA-C in the training dataset than those of HLA-A and HLA-B, we demonstrate that our model can outcompete both pan-allele and allele-specific models for HLA-C alleles in the context of limited number of training data.

### MATHLA enables depiction of a variety of HLA-ligand binding patterns

**Fig. 4:**
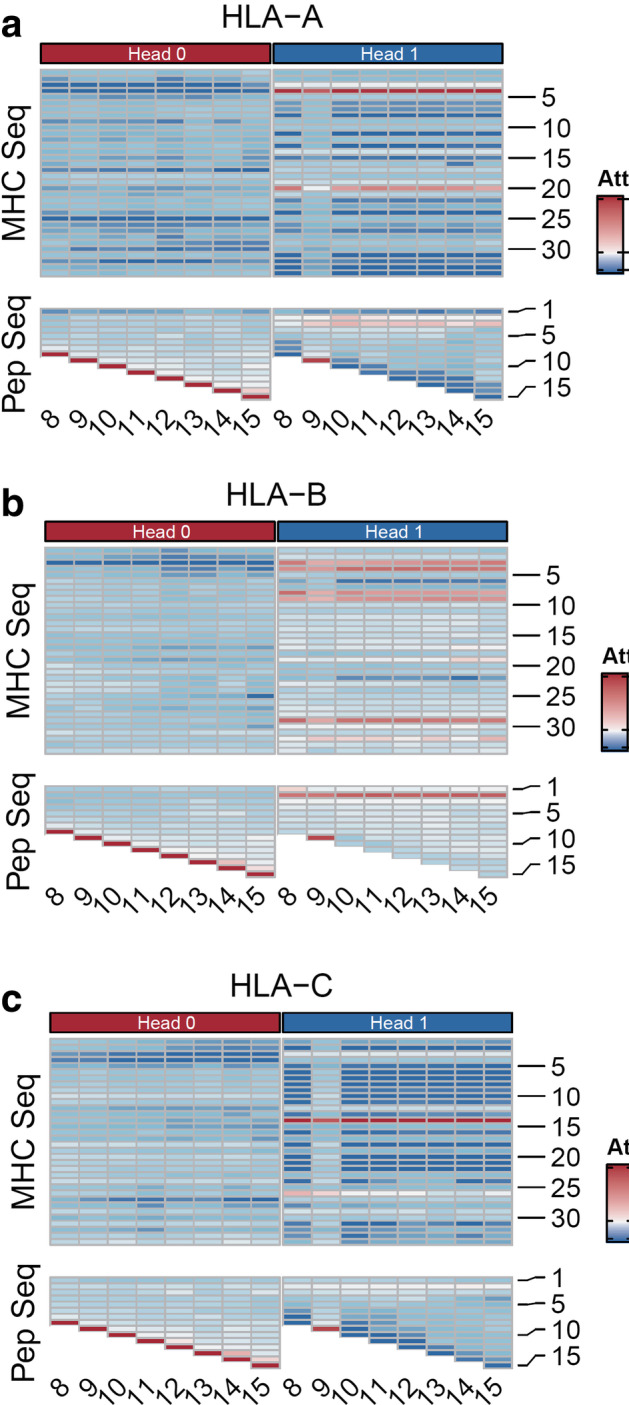
Heatmap of weight scores of two heads (head 0 and 1) in the attention model for both HLA pseudo-sequences and peptide sequences. Weight scores of the test data associated with HLA-A, B and C supergroups are displayed separately. Each row is corresponding to the position of amino acid residue on HLA pseudo-sequence and peptide sequence. Each column corresponds to peptide of different lengths (8aa to 15aa)

To better understand the characteristics of model integrating bi-directional LSTM and multiple head attention mechanism, we investigated the attention weight scores corresponding to different supertypes of HLA class I molecules as well as ligands with different lengths. Previous motif analyses of HLA ligands revealed that the residue at the most C-terminal end was most likely to have recurring amino acids than other positions [25], which was confirmed by the consensus dominant weight score for the last residue of peptide sequence in the head 0 vectors across all three HLA class I supertypes (Fig. 4). On top of the consensus pattern in the head 0 vectors, we observed more diversified patterns from the head 1 vectors. First, we found the weight distribution for peptide of 9 amino acids was distinct from peptides of all other lengths, in which the 9th position on the peptide is weighted dominantly. This pattern is consistent with previous finding that C-terminal residue is more important for the binding of 9-mer peptide than longer peptides [26]. Second, the attention weight scores of the second or third position on peptides bound by HLA-A and B are consistent with another known motif, while the corresponding positions for HLA-C ligands accounts for much less weights. This distinguished weight pattern of HLA-C ligands might explain why our model achieves greater advantages over other tools for HLA-C-peptide prediction. Collectively, our model demonstrates incorporating multiple head attention mechanism into LSTM network can capture HLA-supergroup-specific and peptide-length-specific information that enhances the robustness of MATHLA in HLA-ligand prediction.

## Discussion

Machine learning based HLA-peptide binding prediction have been undergoing rapid development in the past few years owing to the breakthrough of deep learning algorithm and emergence of large-scale mass spectrometry data. Meanwhile, prediction tools have been genuinely deployed in more and more clinical studies than before, due to the booming of cancer immunotherapy. The current criteria for selecting optimal tool in a real-world project mostly rely on the number of supporting alleles and average prediction accuracy measured by different metrics like AUC. However, in regard to prediction accuracy over individual alleles, there is no tool whose prediction accuracy is unanimously higher over all the individual HLA alleles. This fact has posed several questions about HLA-peptide prediction on top of previous criteria like average prediction performance. First, for any given individual patient subject to target identification for cancer immunotherapy, how to select from a variety of existing tools the most accurate one for the given HLA alleles of this patient? This question becomes even more urgent and critical for patients carrying rare HLA alleles or alleles with limited amount of experimental data. Second, for longer ligands (11mer to 15mer) whose prediction accuracy is relatively lower, how to further improve the prediction accuracy given the fact the increasing mass spectrometry data alone can only provide limited prediction power.

## Conclusions

Our model integrating bidirectional LSTM and multiple head attention mechanism has addressed these two questions by not only achieving prominent advantage in prediction accuracy for the HLA-C alleles but also attaining better prediction power for longer class I HLA ligands. Our work shows that advanced architecture of deep learning can provide an interpretive model to further improve and understand HLA-peptide binding prediction. We envision that introducing other alternative methods, such as self-attention mechanism and word2vec model, can provide better peptide representation to further improve prediction accuracy. Our framework will certainly benefit T cell-based vaccine development in treating cancers as well as for prevention of infectious diseases.

## Data Availability

The software is freely available at https://github.com/MATHLAtools/.

## Abbreviations

HLA: Human leukocyte antigen
LSTM: Long short-term memory
AUC: Area under the receiver operating characteristic curve
CNN: Convolutional neural network
MSE: Mean squared error
